# “Brain Injuries Affect Everything:” Long-Term Caregiver Perspectives on Medical and Educational Needs Following Inpatient Rehabilitation for Pediatric TBI

**DOI:** 10.3390/bs16071122

**Published:** 2026-07-05

**Authors:** Jennifer P. Lundine, Nicole Viola, Christine Koterba, Angela Ciccia

**Affiliations:** 1 Department of Speech & Hearing Science, The Ohio State University, Columbus, OH 43210, USA; 2 Division of Clinical Therapies & Inpatient Rehabilitation Program, Nationwide Children’s Hospital, Columbus, OH 43205, USA; 3 Department of Pediatric Psychology and Neuropsychology, Nationwide Children’s Hospital, Columbus, OH 43205, USA; christine.koterba@nationwidechildrens.org; 4 Communication Sciences Program, Department of Psychological Sciences, Case Western Reserve University, Cleveland, OH 44106, USA; amh11@case.edu

**Keywords:** traumatic brain injury, pediatric, caregiver, qualitative

## Abstract

This qualitative study incorporates caregiver perspectives to identify their (1) experiences with medical and educational supports for their children with chronic TBI following inpatient rehabilitation and across the recovery trajectory and (2) recommendations to improve service provision for young people with TBI. Nineteen caregivers of children with complicated-mild-to-severe TBI participated in semi-structured virtual interviews. Participants were from a large Midwestern U.S. city. Researchers used reflexive thematic analysis, incorporating an experiential orientation and a deductive approach. Standards for Reporting Qualitative Research guided this process. Children were an average of 5.2 years post-injury, and age at injury ranged from 2.6 to 18.0 years, providing depth of caregiver experiences discussed in interviews. Four primary themes were identified: (1) TBI leads to lasting changes in the child, (2) the healthcare environment is overwhelming, (3) TBI forces a shift in caregiver responsibilities, and (4) school challenges persist over time. Caregivers generated concrete, experience-based recommendations, highlighting the need for increased support, resources, and education in specific areas following pediatric TBI. By centering caregiver voices across recovery, this study underscores their unique expertise in identifying system-level gaps and informing the development of interventions, services, and policies that better support children with TBI and their families over time.

## 1. Introduction

Traumatic brain injury (TBI) in children represents a significant public health concern and a leading cause of morbidity and mortality (Haarbauer-Krupa et al., 2024; Taylor et al., 2017). Following TBI, children can experience a range of impairments both in the acute recovery period and for months to years later (Brenner et al., 2021; Dahl et al., 2024; Fuentes et al., 2018). As children progress through development, injury-related impairments may emerge or evolve depending on the age of injury and ongoing developmental milestones (Agrawal et al., 2025; Kurowski et al., 2023). While the nature of injury-related deficits has been well established, effective interventions to improve outcomes remain limited (Laatsch et al., 2007, 2020). Existing intervention research inadequately addresses the considerable heterogeneity within the population of youth with TBI that coincides with the complexity of simultaneous development and complicates clinical trials (Agrawal et al., 2025; Covington & Duff, 2021).

Family-centered care is a widely accepted practice within pediatric rehabilitation (Botchway et al., 2022) that recognizes the impacts of pediatric TBI on families (Allonsius et al., 2021; Anke et al., 2019; Jenkin et al., 2022) due to its sudden onset and chronic long-term implications. While decades of research have identified the unmet needs of youth with TBI (e.g., Alessi et al., 2026; Dasarathi et al., 2011; Fuentes et al., 2018; Slomine et al., 2006), until recently, pediatric TBI research and intervention development have not often included the perspectives and experiences of families. Furthermore, existing studies may focus narrowly on mild TBI (e.g., Graves et al., 2020; Minney et al., 2019), or on a specific topic such as community re-integration (e.g., Diener et al., 2022) or emotional loss (e.g., Yehene et al., 2021). In the few studies that have examined caregiver input, they have identified financial strain, lack of mental and emotional support, and insufficient resources as common barriers (e.g., Diener et al., 2022; Graves et al., 2020; Minney et al., 2019; Yehene et al., 2021). These studies include recommendations such as support groups, education modifications, and care coordinators (Dahl et al., 2024; Graves et al., 2020; Quatman-Yates et al., 2022; Slomine et al., 2006; Yehene et al., 2021). Caregivers highlight resources they view to have a positive impact post-TBI, including support through social media, return-to-school programs, and school assistance with academic needs (Dahl et al., 2024; Diener et al., 2022; Minney et al., 2019; Quatman-Yates et al., 2022; Sullivan et al., 2020). When taken together, caregivers consistently cite the need for wraparound support that does not just address the child in isolation but rather addresses their needs across multiple contexts and with multiple stakeholders (e.g., caregivers, teachers, community members). What remains missing, however, is giving caregivers the opportunity to reflect on their acute and inpatient rehabilitation care and consider how it fits their current needs in the chronic stage, years after their child’s injury. Understanding acute and chronic issues is essential to develop interventions that truly address the longitudinal nature of TBI care, which is not well represented in the literature to date.

To improve the long-term care of youth with TBI, caregiver perspectives across the continuum of recovery may identify persistent needs and strategies that align with the actual lived experience of having a child with a TBI. Using semi-structured interviews, this qualitative study aimed to describe caregiver (1) experiences with medical and educational supports for their children with chronic TBI and (2) their recommendations to improve service provision to address chronic needs for young people with TBI. Because a child’s needs post-TBI change across development, this study included caregivers of children with a wide range of time since injury so that caregivers’ commentary could focus on their child’s and family’s needs years post-injury.

## 2. Materials and Methods

### 2.1. Participants

The study was conducted in accordance with the Declaration of Helsinki and approved by the Institutional Review Board of [OSU STUDY00000506, approved 17 October 2019] and [Nationwide Children’s Hospital 2019N0035, approved 22 October 2019]. Informed consent was obtained from all subjects involved in the study. Families were recruited from an urban, pediatric medical center in a Midwestern U.S. city. Eligibility criteria included being a primary English-speaking caregiver of a child who (1) sustained a post-perinatal mild-complicated to severe TBI at or before 18 years of age, (2) was between 1 and 25 years of age at enrollment, and (3) had an injury at least 12 months before enrollment. TBI severity was documented through medical record review (lowest recorded post-resuscitation Glasgow Coma Scale [GCS (Teasdale & Jennett, 1974)] scores of 13–15 with evidence of intracranial lesions = complicated-mild, 9–12 = moderate, and 3–8 = severe). Exclusion criteria included (1) presumed child abuse as the injury mechanism, or (2) pre-injury history of autism spectrum disorder, developmental delay, severe language/cognitive impairment, or neurological disorder. Given a partial HIPAA waiver, purposeful sampling included 108 letters mailed to families of children who met inclusion criteria from the internal inpatient rehabilitation database (injuries sustained from 2009 to 2018). Sampling from the inpatient rehabilitation program allowed for recruitment of families who were reasonably expected to have had similar levels of medical and allied health interventions and to ensure inclusion of persons with various lengths of time since injury. Interviews were completed between fall 2019 and spring 2020. Researchers aimed to recruit at least 15 caregivers for this study, consistent with a systematic review guiding qualitative studies with individual interviews (Hennink & Kaiser, 2022).

### 2.2. Semi-Structured Interviews

Following verbal consent, trained research assistants conducted individual interviews using an online conference-call platform that recorded calls. Following each interview, participants received a $50 gift card as compensation. Research assistants included two speech-language pathology graduate students and one research coordinator with a Psychology undergraduate degree. The first and third authors, both PhD-level rehabilitation clinical researchers, trained interviewers, provided feedback, and reviewed recordings of initial interviews for feedback and quality control.

Study reporting is guided by the Standards for Reporting Qualitative Research (SRQR; O’Brien et al., 2014). Interviews focused on lived experiences of caregivers whose children received medical and rehabilitation care following TBI. Consistent with the semi-structured interview format, interviewers used a “road map” with specific questions to elicit responses and allow for the opportunity to pose follow-up questions. Interview questions were specifically structured to elicit caregiver perspectives focusing on the family’s experience within the medical system, return to home and community, and return to school. Questions were grouped based on timing post-injury: immediately after injury, after medical intervention/discharge, and present day. Caregivers were also asked about ways to improve service delivery following pediatric TBI. The interview guide is available as Supplementary Material. Interviewers took notes in vivo and later transcribed recorded interviews. When interviewers and researchers reviewed the final transcripts, they identified no new ideas being introduced, thus suggesting data saturation had been reached. A second research team member (i.e., undergraduate speech-language pathology student) verified each transcription by listening to the interview while reading and editing the transcript. Discrepancies were corrected, and the first author resolved questions.

### 2.3. Reflexive Thematic Analysis

Interviews were analyzed using reflexive thematic analysis, a progressive and iterative process (Braun & Clarke, 2006, 2019). Reflexive thematic analysis recognizes the researcher’s role and potential bias in creating initial interview questions and identifying and interpreting meaning across the interviews (Braun & Clarke, 2019; Gough & Madill, 2012). Using this model, we recognized and incorporated our clinical research perspectives that may shape this work. The first, third, and last authors on this manuscript led this research. The first and last authors are speech-language pathology clinical researchers with 10+ years of direct experience with youth and families following TBI. At the time of the analysis, the second author was a PhD candidate and speech-language pathologist with 4+ years of direct clinical experience with individuals post-TBI. The third author is a board-certified pediatric neuropsychologist with 10+ years of clinical and research experience in pediatric rehabilitation.

Transcripts were uploaded into NVivo 12 for coding (QSR International Pty Ltd., 2018). Thematic analysis was shaped by Braun and Clarke’s 6-phase process (Braun & Clarke, 2014, 2019), while not necessarily linear (Byrne, 2022), steps were revisited as researchers examined and considered data and how it related to themes. Interviews were considered from an experiential orientation, meaning that the participants’ thoughts and experiences were interpreted as reflective of personal states. Additionally, researchers adopted a deductive approach, meaning researchers considered the interview questions and time-since injury to guide initial code creation (Saldaña, 2021). Researchers also considered the extant literature on gaps in care for youth with TBI during initial code creation. Coding was first completed independently by the first and second authors, who read transcripts and took notes regarding caregiver perspectives as related to service provision, satisfaction, and recommendations in both the medical, home, and education settings. The initial saturation assessment plan (Moore et al., 2026) established by the authors sought to identify when interviews no longer brought new information to the research. First and second authors initially reviewed interview notes and initial codebooks from the first 15 interviews. At this point, new information continued to emerge from interviews. The decision was made to add approximately 5 additional interviews. Four additional interviews were scheduled, completed, and coded. Following the coding of these interviews, researchers determined that analytical redundancy was present in interviews and the established plan for data saturation had been reached. The first and second authors then met weekly (spring–summer 2022) to discuss coding and revise codes iteratively. Once the first and second authors had combined quotes and agreed upon the first versions of codes, they discussed with the third and fourth authors until the next iteration of themes and subthemes were organized and established. A final reorganization of coding occurred prior to dissemination as the manuscript authors regularly discussed, collaborated, and refined their interpretation, and generated themes related to caregiver experiences and long-term needs. To enhance trustworthiness, interviews and select quotations for each theme and subtheme were re-read by all authors to affirm that themes generated by the researchers reflected the participants’ perspectives, across injury severity, age, and time since injury.

## 3. Results

### 3.1. Sample Characteristics

Nineteen caregivers (90% mothers) completed interviews, ranging from 25 to 97 min (M = 50 min). All children had TBI-related hospitalizations requiring inpatient rehabilitation. Table 1 includes demographic and injury-related information. Table 2 includes TBI severity, gender, age at injury, length of stay, and time since injury for each participant.

### 3.2. Themes

Based on caregiver responses, researchers identified four major themes with associated subthemes focusing on the caregiver experiences of medical and education service provision and needs: (1) TBI leads to lasting changes in the child, (2) the healthcare environment is overwhelming, (3) TBI forces a shift in caregiver responsibilities, and (4) school challenges persist over time. Caregivers discussed challenges related to gaps in service delivery and support needs, as well as positive experiences. Example quotes are shared for each theme and subtheme (Table 3). Exact quotations are indicated with quotation marks and noted with an ellipse to remove repetitions, revisions, or to indicate shortening of a quotation. Pronouns in individual quotes were kept as spoken to reflect the caregivers’ representation of their child. Quotes are followed by participant identification number in parentheses. Themes and subthemes are presented below with caregiver recommendations at the end of each theme. Caregiver-generated recommendations related to each theme are shown in Table 4.

### 3.3. TBI Leads to Lasting Changes in the Child

Following their child’s injury, caregivers described grieving the loss of who their child was before their injury and emphasized that these feelings persisted and evolved over time. They specifically struggled with and noticed physical, emotional/behavioral, and cognitive changes. For many, the lack of critical resources (e.g., providers to contact when issues arose after discharge), education, and support made it even harder to adjust to a “new normal” post-TBI.

#### 3.3.1. Physical and Accessibility Challenges Create More Limitations

Physical challenges ranged from navigating mild vision and hearing difficulties to battling physical impairments that impacted walking and/or completing activities of daily living. While caregivers were aware of the physical changes that resulted from their child’s TBI, the reality of these in everyday life became apparent once they were discharged from hospital care as they tried to navigate their home and community. Physical changes impacted the child’s self-esteem and their ability to engage in their environment. Caregivers were concerned about the reactions of others and were unsure how to address these in real time. Physical changes also caused accessibility barriers that made it hard for their child to participate in home and community activities. For example, several caregivers shared that they were unable to make their home accessible when their child could not walk independently or climb stairs. In some cases, this made it impossible for their child to sleep in their own room or take an actual bath or shower.

#### 3.3.2. Families Are Unprepared for the Significance of Emotional/Behavioral Changes

Many caregivers did not feel equipped to deal with their child’s injury-related depression, anxiety, and post-traumatic stress that persisted long after returning home. Several caregivers discussed challenges helping their children manage post-injury changes, not wanting their children to view themselves as weak. Caregivers also expressed concerns related to bullying after the injury, changes in social status due to activity limitations and physical impairments, and the child feeling ashamed for being different. Collectively, caregivers struggled to differentiate between “normal” emotional fluctuations and TBI-related changes.

Caregivers said hospital mental health services for their child were helpful but minimal, and that the time in the hospital was primarily focused on physical recovery, rather than emotional/behavioral functioning. The need for mental health services throughout recovery was a common topic, and many participants felt that while mental health support was vital, it was often hard to find. This was especially true once families were home. Several caregivers independently sought mental health support for their child after discharge, which took weeks to months to access. Others in rural areas were unsuccessful despite their best efforts. Additionally, caregivers lacked education and training to handle personality and behavioral changes, including new physical and verbal aggression.

#### 3.3.3. Cognitive Changes Affect Everyday Life

Many caregivers reported post-injury cognitive changes related to memory, perseveration, attention, and executive functions and noted how these changes adversely impacted their child. Some children succeeded with environmental cues while others were unable to pay attention and recall information even with external supports. Some caregivers felt as if their child’s maturity had regressed. Caregivers worried about how cognitive deficits would impact their child’s ability to live independently in the future and did not feel they had resources to support long-term needs. For example, several felt that while their child could handle day-to-day responsibilities, they struggled with higher-level skills needed for increasing levels of expected independence (e.g., financial skills). Overall, caregivers described feeling ill-prepared to address the cognitive effects of their child’s TBI both at the time of the interview and when projecting into the future.

#### 3.3.4. Caregiver Recommendations Based on Lived Experience

Caregivers shared recommendations they believed would help them cope with physical, emotional, and cognitive changes they discussed with the research team. Specifically, many caregivers cited TBI research and education as being vital. They felt that increasing TBI awareness could help increase community acceptance. For example, one caregiver stated:

Resources. Education. If there were more resources for TBI, like there are for people who are Deaf, or blind, or with cancer, people would better understand. I think it would put people with TBI more at ease…if [other] people better understood them. The only way to better understand [people with brain injury] is education and resources (P23).

To address emotional changes, caregivers expressed needing more mental health services with providers trained to work with pediatric TBI. Caregivers requested more frequent mental health support for their child while in the hospital and a list of local providers to find treatment more easily after discharge. Caregivers discussed that all family members needed support aimed at adjusting to life with a loved one with TBI “…because it’s hard for everybody (P70).” In addition, caregivers in rural locations suggested that they would benefit from consistent school-based mental health services designed for children with TBI in a safe and supportive school environment. Many expressed that support groups with caregivers of children with TBI would be beneficial to have an opportunity to connect to those with similar experiences. In the absence of groups, caregivers suggested finding support through social media platforms. Finally, to support cognitive recovery and independence, caregivers suggested a community-based class to address daily living skills, such as managing finances.

### 3.4. The Healthcare Environment Is Overwhelming

Even many years after their child’s TBI, caregivers recalled being overwhelmed in the early days after their child’s injury. A rapid influx of information, concerns about inadequate staff training, and confusion about community support contributed to caregiver stress that reduced their confidence in the care their child was receiving and the care they would need to provide after discharge.

#### 3.4.1. Education Is Essential—But Timing Is Critical

Caregivers highlighted that their child’s immediate medical needs were the primary concern in the hospital. Many could not fully benefit from education during their inpatient stay because they were overwhelmed with their child’s recovery, both acutely and during times of intensive medical needs. Caregivers struggled to retain information when it was provided in only one format or when they perceived they were provided information only once. While they appreciated written information, it was difficult to keep track of everything when they were already overwhelmed. Caregivers also reported that it was challenging to ask questions because medical teams and providers changed frequently, and it was hard to speak with providers if they did not come at the same time each day.

#### 3.4.2. Individualized Care Makes a Difference

Caregivers were strongly impacted by interactions with staff members across healthcare settings. The majority described an overwhelmingly positive experience, especially when they perceived their children to be receiving individual or family-focused care. Some, however, experienced negative interactions and felt their child was not a top priority. One caregiver felt that her child had been depersonalized, and another felt that staff members were not adequately trained to treat patients with TBI-related impulsivity and agitation. Concerns related to staff training caused some caregivers to react with hypervigilance, feeling unable to relax when they were not at the hospital watching over their child.

#### 3.4.3. Families Need Help Identifying Resources

Adding to the chaos and confusion, caregivers felt the lack of knowledge and resources, or the challenge of processing the information they had been given, limited their ability to access services before and after discharge. Primary barriers included insurance coverage and approval for services, distance from the hospital, and limited services/providers. Caregivers felt overwhelmed trying to address issues ranging from dental care, psychological support, and even primary care with providers who understand TBI. Families in rural areas and those without consistent transportation discussed challenges accessing services outside of their community. Those in urban areas also struggled to access care because even when specialists (e.g., rehabilitation providers, neuropsychologists) were nearby, few were trained to work with pediatric TBI or there were substantial waitlists for evaluation or treatment. Further complicating matters, both caregivers in rural and urban communities were unsure where or how to find specialists to meet their child’s specific needs.

#### 3.4.4. Caregiver Recommendations Based on Lived Experience

To address challenges in the healthcare setting, caregivers recommended modifying the amount, timing, and manner of education delivery to maximize comprehension amid a chaotic inpatient environment. Many felt they could engage more if hospital staff provided education in multiple formats (i.e., verbal, written, demonstration), repeated key points often, and began education *after* their child was medically stable. Caregivers recommended simplifying medical terminology. They felt prioritized when individualized care came from a consistent care team, which improved communication amongst team members and with the family. Several caregivers noted that TBI-specific staff training could also improve care, particularly in areas where staff may have less familiarity with TBI recovery. Caregivers recognized the impact a positive relationship with their child’s care team had on their ability to engage, practice therapy exercises, and ask questions. Those who received individualized care expressed fewer concerns and recognized when staff went above and beyond to meet the family’s needs. Consistently, caregivers recommended having a dedicated care coordinator to help them navigate and manage TBI-specific resources.

### 3.5. TBI Forces a Shift in Caregiver Responsibilities

As they settled into life after their child’s injury, caregivers discussed that they felt unprepared for their new responsibilities following their child’s TBI. They shared how challenging it was to learn new skills needed to support their child with a TBI at home and in the community.

#### 3.5.1. Discharge Planning Does Not Adequately Prepare Families

Caregivers were overwhelmed by the transition home and were not practically or emotionally prepared for the realities of caring for their child at home. Caregivers noted the difference at home and felt that they could not provide the same level of care as in a medical setting, such as caring for their other children while also meeting the needs of their child with TBI. Many felt that TBI-specific parenting resources were nonexistent and felt unprepared for the reality of caring for their child. Some caregivers described a 24 h practice period to perform all care before discharge, but while nursing was available. They noted that this practice period did not capture the true complexity of being home, where they would need to simultaneously juggle work responsibilities and caring for other children, all while managing care for their child with TBI. In addition, the 24 h period did not fully capture the reality of caring for their child in the family’s home, which would not be fully equipped like the hospital. Upon returning home, some lacked necessary equipment, like shower chairs or ramps. Others discussed necessary home renovations that could not be completed before discharge, for example, replacing carpet with hardwood floors to facilitate wheelchair accessibility.

#### 3.5.2. Children and Caregivers Feel Isolated Once Home

After discharge, caregivers felt isolated and alone. Many caregivers worried about overburdening others and felt that family and friends did not understand their circumstances, resulting in waning support over time. Children also became isolated as they could not socialize, interact with peers or participate in extracurricular activities as they did before the injury. Isolation persisted for years as children’s friends continued to the next life stages while the child with TBI was not yet ready, or unable, to make these same transitions. Caregivers felt responsible, but also expressed feelings of helplessness in knowing how to navigate their own and their child’s feelings of isolation.

#### 3.5.3. Caregiver Recommendations Based on Lived Experience

Caregivers gave suggestions to improve their preparation for transitioning home and living with the unpredictability of a chronic TBI. They recommended at least one home visit and procuring all equipment before discharge to improve their comfort level. They recommended adjusting discharge planning to prepare them for the realities of caring for their child independently at home. Some suggested they should be responsible for caring for all their children and responsibilities during the 24 h exercise (not just the child with TBI) or bringing their child home for the transition activity instead of the hospital-based experience. Then, problem-solving with hospital staff could address issues that arose at home. To address social isolation, caregivers suggested community-based programming for children and families with TBI. Related to feelings of isolation, families again reinforced the value of connecting with other families who had experience with TBI to learn from each other and share experiences.

### 3.6. School Does Not Get Easier with Time

Caregivers highlighted gaps that made their child’s return-to-school difficult and impacted academic and social functioning, including challenges arising over time or persisting years after the child’s injury. Despite not having medical or educational expertise, caregivers felt the heavy responsibility of advocating for their child.

#### 3.6.1. Caregivers Feel Responsible to Bridge the Medical-School Gap

Several caregivers felt responsible for communicating with schools about their child’s injury. However, their lack of understanding of the consequences of TBI and its manifestation within the educational system made it challenging. Many caregivers expressed frustration and dissatisfaction with the implementation of special education or school accommodations following their child’s injury. Medical and school staff often had different goals and did not coordinate efforts to meet the child’s needs, and caregivers felt stuck in the middle. Many caregivers described difficulties having perceived needs met at school, and that these needs persisted long after the injury. In some cases, new issues arose many years after the child’s injury, making it harder for caregivers to help their child get the support they felt was needed. Caregivers also felt school staff did not adequately address social difficulties like making friends and avoiding dangerous peer situations. When issues with academic and social support arose, caregivers were unclear who to contact and lacked support navigating special education. Many caregivers harbored negative feelings toward the school because they perceived that the child’s needs were not being met.

#### 3.6.2. Schools Need More Preparation to Support Students with TBI

Caregivers frequently recognized that school staff did not have adequate training or resources related to TBI. Many felt schools were unprepared and disorganized, which negatively impacted their child. Many caregivers reported struggles at school were not just immediately after injury but also persisted or changed years later. They struggled to understand why schools were not better able to support students with TBI, especially given that schools appeared better equipped to deal with many other conditions that impact academic performance (e.g., deaf, blind, learning disability). Caregivers whose children attended a smaller school/district or had educators with knowledge of TBI shared more positive experiences.

#### 3.6.3. Caregiver Recommendations Based on Lived Experience

To address school needs, caregivers recommended that an advocate in IEP meetings would help ensure their child received appropriate accommodations. They also suggested a school liaison or care coordinator could improve communication between the hospital and school to ensure both teams coordinated efforts and got “on the same page (P93).” Caregivers expressed that it would be helpful to have a single contact to call with any concerns. Finally, they endorsed opportunities for staff education related to TBI and its educational impacts to ensure a successful return to school. For those who had the opportunity, a meeting with hospital and school staff before their child returned to school helped address gaps in knowledge and preparedness but was not sufficient to ensure their child’s needs were met over time.

## 4. Discussion

The aim of the present study was to gather caregivers’ perspectives related to support for their child with chronic TBI across the recovery process and recommendations to improve service provision to address identified needs. TBI in childhood is common (Haarbauer-Krupa et al., 2024) and represents a chronic condition with potential long-term developmental, social, and physical challenges that can be difficult to manage (Agrawal et al., 2025; Kurowski et al., 2023). Research shows that across injury severity and over time, youth with TBI experience unmet and unrecognized needs (Alessi et al., 2026; Dahl et al., 2024; Fuentes et al., 2018; Keenan et al., 2019). Consistent with previous research, caregiver perspectives in the current study highlight persistent challenges related to post-injury impairments, healthcare resources, caregiver responsibilities, and school support.

While these domains of concern have been described previously, this study adds two important perspectives. First, all families included in this study received inpatient rehabilitation following their child’s TBI. While admittedly this sample represents the *minority* of young people with TBI (Greene et al., 2014), it also represents those who are receiving the highest level of family-centered care (Botchway et al., 2022) and transitional support to return to home and school (T. D. Bennett et al., 2013; Rivara et al., 2012). Of youth with TBI, theoretically, these children and their families should be the most prepared and well-resourced to tackle the challenges associated with post-TBI life. And yet, consistent with studies in adult neurotrauma care (Ahmed et al., 2017), even after receiving the highest standard of care, families still reported many areas for recommended improvement. Secondly, this cohort of families represents a group who were, on average, 5.2 years post-injury, with 30% of families having a child who sustained their TBI more than 5 years prior. Despite a wide range of ages at injury and time since injury, researchers identified themes across interviews, such that caregivers shared consistent experiences despite these different timeframes. The fact that caregivers continued to highlight challenges identified in studies of youth with more acute TBI suggests that these issues do not resolve over time and current interventions and supports are not sufficiently addressing their greatest areas of need. In this way, caregiver experiences highlight how much work must still be done and the areas future interventions must target. Specifically, this work emphasizes gaps that exist for youth and families who receive the highest level of rehabilitation care, indicating that the majority of youth with TBI who do not receive this level of care may be experiencing even greater levels of difficulty than those described in this study (Dasic et al., 2022).

Understanding the lived experiences of caregivers can inform researchers, clinicians, and policymakers of the complex factors that impact outcomes after childhood TBI, especially related to service delivery and strategies to minimize long-term challenges (Lundine et al., 2023). Caregivers emphasized needing mental health and other support services, including peer-support groups and professional help navigating new challenges arising both acutely and years later. Consistent with past work, caregivers discussed the importance of education and individualized care for children and families (Botchway et al., 2022; Dahl et al., 2024; Jenkin et al., 2022) and the massive responsibility to educate schools, along with locating providers with expertise in pediatric TBI (E. Bennett et al., 2023; Roscigno et al., 2015; Yoder et al., 2025).

In relation to multiple themes, caregivers reinforced their need for a care coordinator to support their child’s long-term recovery, advocate for them, and connect them to TBI-specific resources. A care coordinator with expertise in brain injury has been reported as a need in other studies (Diener et al., 2022; Lundine et al., 2023; Minney et al., 2019; Quatman-Yates et al., 2022), but findings from this current study reinforce the persistent and significant unmet needs that caregivers feel ill-equipped to manage even many years after a child’s injury. While this study does not include data regarding participants’ use of community resources, previous research highlights that while pediatric TBI is a significant cause of morbidity and mortality, children remain underserved and understudied in terms of rehabilitation and educational services, both in the acute and post-acute period (Glang et al., 2008; Johnson et al., 2022). A scoping review examining outcome metrics for neurotrauma services in major trauma centers (Dasic et al., 2022) identified a general failure in the adequate provision of rehabilitation services across higher-income countries, and more significantly across low-to-middle-income countries. When rehabilitation is limited, families and individuals with TBI are inevitably left to deal with the consequences of an injury on their own. Our hope is that participatory action research such as this can be used to influence policymaking and development of novel interventions to improve funding and resources for youth with TBI after the immediate injury.

The four major themes identified in this work emphasize that years after TBI, caregivers struggle with lasting changes in their child, care-related challenges, difficulties adjusting caregiver responsibilities, and challenges in school. Findings highlight how unmet needs impact the child and family, even years after injury, and ways that care provision could be improved. In the future, caregiver perspectives should be integrated with the perspectives of medical providers and educators to determine the most realistic and productive ways to support families and children with TBI. As the Centers for Medicare and Medicaid Services in the US are now recognizing TBI as a chronic condition (Centers for Medicare and Medicaid Services, 2024), it is essential to ground systems-level change in the real-life experiences of families who live with TBI.

To translate these findings into systemic action, policy reforms can leverage the recognition of TBI as a chronic condition to expand insurance reimbursement for long-term care coordination, ensuring continuous access to specialized resources. Additionally, addressing persistent school challenges requires strengthening Return-to-Learn infrastructure through formalized medical-educational partnerships that relieve the advocacy burden currently placed on families. By formalizing hospital-to-school transition protocols for all injury severities and funding dedicated professionals who are cross-trained in both neurorehabilitation and special education, systems can adequately address individual needs into actionable and specific classroom accommodations. By doing so, a true continuum of care would be established that improves long-term outcomes for youth with chronic TBI.

### 4.1. Limitations

Several limitations should be addressed as this work progresses. While participants in this study provided valuable insights, we recognize perspectives may differ depending on various factors that deserve further exploration (e.g., race, socioeconomic status, parental education, geographic location). In addition, our sample predominantly included caregivers whose children experienced moderate-to-severe injuries, all of whom were admitted to an inpatient rehabilitation unit. While stated as a unique strength of this study, we also note the limitation of this sampling as well. A small minority of youth in the US with TBI receive inpatient rehabilitation (Rivara et al., 2012), so the views expressed in this study may not accurately reflect the experiences of children with milder injuries or those who receive different levels of care. Additionally, our sample included an oversampling of male youth with TBI and female caregivers. While males are more likely to sustain a TBI than females (Taylor et al., 2017), future work should focus on ensuring the voices of families with female survivors of TBI are well-represented. Additionally, it is important to explore male caregivers’ experience of having a child with TBI.

Other than those who received inpatient rehabilitation (including occupational, physical, speech, recreation therapies, hospital-based school services), this study did not collect data about other specific inpatient/outpatient services or community resources (e.g., support groups, state Brain Injury Association helpline support) youth may have received post-injury. Unless caregivers specifically talked about outpatient or school-based services, we cannot make broad statements about the care received. Similarly, we do not have specific data regarding the type of education families received during their hospitalization or information regarding caregiver retention of education and training. Future research should include data from patients’ medical records regarding services received during the inpatient admission along with delivery of education.

### 4.2. Conclusions

Previous research has documented the importance of family members in outcomes following TBI, yet few studies have sought insight from families themselves many years after their child’s injury. This study adds uniquely to the literature by including families who have been living post-TBI for many years, allowing caregivers to reflect upon their experiences. Caregivers consistently discussed short- and long-term challenges that impact the family and child with TBI, emphasizing the chronic nature of brain injury in children’s and families’ lives. Caregiver perspectives provide valuable insights regarding necessary improvements in care provision for children with TBI acutely and throughout the chronic stages of recovery. Caregiver perspectives can ultimately drive change at each level of recovery following pediatric TBI to improve outcomes for children and families. Without systematic inclusion of caregiver perspectives, we miss crucial insights that could meaningfully change long-term care and rehabilitation of children with TBI.

## Figures and Tables

**Table 1:** Demographic, injury, and rehabilitation characteristics of children (n = 19) and caregivers (n = 19).

Demographics	n (%)	Mean	SD	Range
CHILD				
Age at injury (years)	19	11.4	4.6	2.6–18.1
Sex				
Female	2 (10.5)			
Male	17 (89.5)			
Race and Ethnicity				
White, non-Hispanic	14 (73.7)			
White, Hispanic	1 (5.2)			
Black, non-Hispanic	2 (10.5)			
Multi-Racial	2 (10.5)			
Severity of Injury				
Mild-Complicated	1 (5.3)			
Moderate	2 (10.5)			
Severe	16 (84.2)			
Time Since Injury				
1–4.9 years	13 (68)			
5–7.9 years	3 (15.8)			
>8 years	3 (15.8)			
Length of stay (days)	19	53.95	30.83	14.00–111.00
Mechanism of Injury	19			
Motor Vehicle Collision	17 (89.5)			
Struck by object	1 (5.3)			
Fall	1 (5.3)			
CAREGIVER	n (%)	Mean	SD	Range
Level of education				
High School/GED	6 (31.6)			
Associate’s	8 (42.1)			
Bachelor’s	3 (15.8)			
Master’s	2 (10.5.5)			
Age at interview	19	40.15	4.73	32.00–52.00
Relationship to child				
Mother	17 (89.5)			
Father	1 (5.3)			
Grandmother	1 (5.3)			

**Table 2:** Participant identification number with demographic and injury-related information.

Participant ID	Severity of Injury	Gender	Age at Injury (Years)	Length of Stay (Days)	Time Since Injury (Years; Months)
P1	Severe	Male	18	98	1;6
P8	Severe	Male	15	56	2;9
P9	Severe	Male	15	33	2;2
P13	Severe	Male	13	30	3;11
P20	Severe	Male	7	111	8;7
P23	Severe	Male	13	103	1;7
P25	Moderate	Male	12	25	1;7
P27	Moderate	Male	11	30	6;6
P32	Severe	Male	12	40	1;2
P56	Severe	Male	4	64	8;9
P57	Severe	Male	4	54	8;6
P59	Severe	Male	4	15	4;1
P63	Mild-Complicated	Male	2	26	7;10
P64	Severe	Male	15	102	4;6
P70	Severe	Male	13	14	5;4
P78	Severe	Female	9	61	3;10
P85	Severe	Male	16	46	2;9
P93	Severe	Female	10	44	1;8
P98	Severe	Male	17	73	1;0

**Table 3:** Key quotes from caregiver interviews (participant ID noted in parentheses following each quote).

Theme	Subtheme	Key Quotes About Service Delivery and Support
**1: TBI Leads to Lasting Changes in the Child**	1.1: Physical and Accessibility Challenges Create More Limitations	He had to wear a neck brace for three months. That really affected him a lot because…he just couldn’t move, and it just really upset him. Everybody was staring at him (P9). It was a mess trying to get him a wheelchair. [We went to] ball games ‘cause his brother plays sports. [It was hard to navigate] going through the gravel or someone closed the gate in our face to make us go around (P63).
	1.2: Families are Unprepared for the Significance of Emotional/Behavioral Changes	The worst is him adjusting to the disability… he was athletic and he was very popular. He is an outdoor kind of kid [and now] he just sits in his room and cries sometimes because he can’t play football anymore, can’t wrestle, he can’t do anything he did before (P23). I think [he] is just kind of tired of being pissed off all the time,…but he doesn’t want to talk to anybody about it (P9). She is not as open to friends, she always feels judged. She is emotional, moody, anxious (P93). [His hospital stay] was pretty much focused on him being able to get up, being able to walk and all that kind of stuff, but it was not focused on his actual mental well-being (P27). It’s hard to find mental health services in [our county]. I think there’s a shortage…that’s how it is in these little rural areas, we just don’t have the mental help that a lot of these children need (P78). They didn’t prepare me very much for the outburst. They said, “Be careful because he does have outbursts,” but they didn’t prepare me that he might physically harm me or himself (P20). I think he did go through shame, like something’s wrong with him. He knew he was different, and he didn’t want to be different. Like he just kept saying I just wish this never happened (P57). I would say he is probably more prone to some depression and/or anxiety than beforehand, but again how do you know (P85)?
	1.3: Cognitive Changes Affect Everyday Life	The TBI is the reason why he forgot to write down that he had homework yesterday (P13). [He needs] constant verbal cues (P27) [Her] memory is horrible…if you’ve ever seen…[the movie] *Groundhog’s Day* where it’s like you hear the same story ten times in a day, that’s [her] (P93). He can’t stand to be distracted. So when [his classmates] are making a lot of noise he feels like he can’t concentrate and focus (P63). He’s thirteen but cognitively he’s like a seven-year-old. He’s many years behind. (P56). He knows how to work, he knows the working thing, he can clothe himself, he knows how to cook, laundry, clean, he knows all that stuff. But when it comes to finances? He doesn’t understand that. When it comes to his medical? He doesn’t understand that (P20).
**2: The Healthcare Environment is Overwhelming**	2.1: Education is Essential—But Timing is Critical	You’re giving me stacks of paper instead of verbally telling me. I can’t sit and read through all of that when I already have enough on my plate (P23). When your child first gets admitted and you go to the [intensive care unit] you have so many people in your face and there’s just people everywhere and you don’t know if your kid’s gonna live or die. And they’re talking to you and all you hear is noise. But then once you move to a different floor then people stop coming. And then it’s like…you may have said something a week ago, but I didn’t hear you because my kid was on a vent. We didn’t know if she was gonna live. So, I feel like it’s hard because everyone throws themselves at you, but then that’s it. They never come back. And so then it’s like ‘oh maybe I remember that, maybe I don’t, I don’t know.’ It’s just, you’re kind of in a fog at that point and you don’t really, you don’t hear anybody ….You’re so focused on one thing, not what they’re trying to bring to you. When your kid is maybe more stable, then they can bombard you with the information you need, not when you’re not able to pay attention… (P93).
	2.2: Individualized Care Makes a Difference	I remember holding him and…I was trying to keep him from ripping his IVs out…two hospital staff [members] came in and was like, “what do you want us to do?” [They were] standing there trying not to help…I remember breaking down in tears after that because I felt like they didn’t know how to handle it and I was just trying to keep him safe (P63). There needs to be a whole lot more training. If they’re sitting in my daughter’s room, they should not be not paying attention to her…She almost fell out of bed multiple times, and they were sitting right next to her (P93). I remember when I was there that was my biggest complaint, was feeling that [the doctor] depersonalized my son…like he was a case and not a person (P8). I remember there was a nurse that when my son was first going through the stages of brain injury he would get really agitated and would scream and holler and he would be bored all the time so the one nurse would take him out to the desk and have him sit there in his chair and pretend he was working and have him type up stuff to try and keep him busy (P56). What I really loved was that they found a way to not only care for [him] and you know take care of his needs and stuff, but they found a way … to take care of [my needs] so that I could take care of him. …They found a way to work with us as a family (P59).
	2.3: Families Need Help Identifying Resources	[Insurance decides] how successful your recovery is. Basically, how much you can afford to spend or not work so you can give time to your child. I think it’s not a fair system. I don’t think it’s the same for all (P64). There was a difficult point when he first came out of his coma, he currently wasn’t meeting the criteria to go to rehab… I don’t think it was the doctors, it was the insurance…Dollars meant more than the patient (P64). We started traveling an hour and a half to a different therapy place that was very helpful for him but once he started school it got to be too much to go to school all day and then travel an hour and a half for therapy (P63).
**3. TBI Forces a Shift in Caregiver Responsibilities**	3.1: Discharge Planning Does Not Adequately Prepare Families	When you get home, you’re doing it the whole time but you also have other responsibilities at home too. Like more than just that child. So maybe 24 h at home or with a sibling spending that 24-h at the hospital too. … Mama can’t pay attention to you 24-h [a day] like the nurses did (P20). When you’re trying to take care of your kids and get a job and this and that … everything else tends to fall apart and that’s just how I feel, like everything was just falling apart (P25). Her bedroom was upstairs, so we had to take our dining room and turn it into a temporary bedroom for the first few weeks she was home…we had carpet and had to take that up completely and put in hardwood [floors] so it was easier for her to transition (P93). [The hospital] was two and a half hours away from our house. They didn’t do a home visit or anything. So bringing him home I remember thinking, “Okay this house isn’t set up for a wheelchair, he can’t even walk…” I just felt we really weren’t prepared to bring him home (P56).
	3.2: Children and Caregivers Feel Isolated once Home	I think I should have incorporated more of my friends and family, but I was just thinking like unless they volunteer, I’m not gonna put anything on someone else (P27). Although we lived in a very supportive community…we’re four years out now and [his friends have] all gone to college and [my son] has not gone to college so now he doesn’t have a social group (P57). I don’t even know where to call to find something for this kid to do. I can’t just send him to a [recreation] center, I can’t just send him to the YMCA, like, there’s not enough for them to do. There needs to be some type of program or resources. He just sits in his room and plays video games all day because we don’t know what to do (P23).
**4: School Does Not Get Easier with Time**	4.1: Caregivers Feel Responsible to Bridge the Medical-School Gap	I felt that I really had to hold them accountable as far as following his IEP… I just felt like I had to stay on the school too much to get stuff done that I can’t fall, I can’t relax because I’m constantly reminding you guys that you’re not doing this or this needs to get done, so I have to be on top of it because you guys are letting it fall through (P27). I don’t know if the preschool teachers were really equipped with what they needed. Where we were, that was the only preschool in that town. I wish I would have talked to them maybe a little bit more, to see if they know how to take care of him (P57). These are the guidelines set by the care team, why [is the school] not required to follow them?…[After the school re-entry meeting] we get this big packet of recommendations and we can take it to school all we want but if they’re not gonna follow it and there’s no one there to enforce it, I mean that’s where we struggle. Her accommodations aren’t getting met and we don’t have anyone to contact to see how to get it moved forward (P93). It’s just school districts give pushback from what doctors recommend, to what the school district thinks the kids need (P93). [He] finally spoke up and told a counselor at [school] that he was getting bullied…that was the first time he actually ever spoke up and said “Hey I’m having problems with another student, can you please help me out?” and she actually turned around and told him “no” and she knew what was going on with him and she knew that he had a TBI and all this and really did not care at all (P70).
	4.2: Schools Need More Preparation to Support Students with TBI	I feel like there is not enough resources for people with TBI, nor is there enough education…they have a school for the blind, they have a school for the Deaf, they have all types of schools for people and kids with disabilities, but when it comes to TBI I feel like they throw them out to the wolves. They put them back into these regular schools, public schools they attended before their injury. They throw them out to the wolves. There are no resources. The schools are not educated enough. They do not take it serious enough either (P23). [What improved our return to school] was me going in and talking with all the teachers. We actually had a meeting at [the hospital] before that too …where the special needs administrator came into a meeting. That was very helpful (P32).

*Note.* Exact quotations from participants are edited for comprehension (e.g., brackets provide context when needed), and noted with an ellipse to remove repetitions, revisions, or to indicate a shortening of a quotation. Pronouns in themes/subthemes were changed to be maximally inclusive, but those expressed in individual quotes were kept as spoken to reflect the caregivers’ representation of their child.

**Table 4:** Themes and Associated Caregiver Recommendations.

Theme and Subthemes	Caregiver Recommendations
Theme: TBI leads to lasting changes in the child	Increase mental health services for children and families from providers with experience in TBI Provide more frequent visits from mental health providers in the hospital Provide a list of mental health providers in their area upon discharge from the hospital Deliver more school-based mental health services Increase parent support groups-in person, through social media, or online Create community-based life skills classes
Physical and accessibility challenges create more limitations Families are unprepared for the significance of emotional/behavioral changes Cognitive changes affect everyday life	
Theme: The healthcare environment is overwhelming	Present education in multiple formats (i.e., verbal, written, demonstration) Repeat education multiple times Begin education later in hospital stay when child is medically stable Be mindful of medical terminology and ensure caregiver understanding Provide a consistent care team whenever possible Provide additional TBI-specific training for staff (e.g., agitation, behavior management) Establish a care coordinator to help navigate resources during and after hospital stay
Education is essential—but timing is critical Individualized care makes a difference Families need help identifying resources	
Theme: TBI forces a shift in caregiver responsibilities	Prioritize a home visit during hospital stay Order all necessary equipment prior to hospital discharge Deliver more realistic therapy activities that mirror the home environment Provide a 24-hour test run for caregivers to practice solo care of their child
Discharge planning does not adequately prepare families Children and caregivers feel isolated once home	
Theme: School does not get easier with time	Assign a family/child advocate for IEP meetings Establish a school liaison or care coordinator to help improve communication between school and medical personnel Increase school staff education on TBI
Caregivers feel responsible to bridge the medical-school gap Schools need more preparation to support students with TBI	

## Data Availability

The data that support the findings of this study are available from the corresponding author upon reasonable request.

## Supplementary Materials

The following supporting information can be downloaded at: https://www.mdpi.com/article/10.3390/bs16071122/s1, Interview Guide.
