# Supplementary material for: “Brain Injuries Affect Everything:” Long-Term Caregiver Perspectives on Medical and Educational Needs Following Inpatient Rehabilitation for Pediatric TBI
Source: Behav Sci (Basel). 2026 Jul 5;16(7):1122. doi: 10.3390/bs16071122 (PMC13403691; doi:10.3390/bs16071122)
Supplement: Supplementary file 1 [file behavsci-16-01122-s001.zip › behavsci-4350575-supplementary.pdf]

Semi-structured interview prompts

Questions about Medical Care and Immediately Going Home

- Tell me about your child.
- Tell me about your child's injuries.
- Tell me briefly about when you got to the hospital. What happened next? How long were you there?
- Tell us about preparation to return home.
  - What did you feel well prepared to handle?
  - Were there any areas that you felt less prepares?
  - Did anything surprise you about the process?
- Do you have any recommendations to improve the process of returning to home and school?
  - What advice would you give to other families who were in a similar situation?
  - What advice would you give your child's care team (doctors/nurses) and educational team (teachers/therapists)?
- Tell me about your experience with the health care system as it relates to your child's care.
- Tell us about the training and education provided to you about your child's injury.
- We've talked a lot about the hospital stay and just getting home, what would you say as one major thing you could say about that period?
  - It could be a single word, or a few sentences-what is your biggest takeaway?

Questions about later, further down the road

- Tell us about your child's experience in school.
  - Any special services or help your child received after his/her TBI?
  - Were you happy with any services your child received?
- Tell us about any issues arose at school or home that you didn't know how to deal with or you didn't know who to reach out to about.
- Tell us about any situations where you felt confident about how to deal with them or to whom you could reach out for help.
- Who were the people that worked with your child after you left the hospital?
- Tell us about follow-up services for your child. Did you feel that you were getting help for all the things you needed?
  - Were there types of people that you wish you could've worked with but did not for some reason?
  - Or did you did believe that you should've had certain types of care that you did not or could not receive?
- If you experienced certain challenged that were not addressed, why do you think those things weren't addressed?
  - Did you face any of these issues…
    - Financial (insurance, work requirements/employment, can't leave work, bills from hospitalization)
    - Physical (transportation, time, distance from the hospital, childcare, competing needs of other family members, too many appointments/scheduling didn't fit my needs)

        - Psychosocial (stress, depression, sleeping difficulties, emotional support)
        - System (didn't know who to call, couldn't get a referral, professional willingness to provide support)
    - Tell us some things that you believe would've helped to fix this situation.
- Tell us about the positive impressions you have regarding all the care your child received, including hospital, rehab, and school.
- Tell us about the gaps you perceive in the care your child received, including hospital, rehab, and school.

Recommendations

- If you could do anything, tell us about any changes you would make to the care your child received in the hospital, rehab, or going back to school.
- What are some ways that the system could be improved to help families who have children who experience a TBI?
    - Would a person have helped? Would a place where you could go to locate resources help? If so, what do you think that would've looked like? Would you prefer a "thing" that could help?
- If things were really good, what things do you think made the care your child received so successful?
- We've talked a lot about your experiences as you moved further from the injury. What would you say as one major thing you could say about that period?
    - It could be a single word, or a few sentences. What is your biggest takeaway?

Questions about the Present

- Right now, do you think your child is the same or difference since their brain injury?
    - Tell us about any changes you perceive.
- What keeps you up at night when you think about your child?
- Do you perceive a change in your child's future life plans as a result of his/her injury?

Extra Questions

- Tell us about insurance and the role it played in your child's medical care.
